# Stability Study of Synthetic Diamond Using a Thermally Controlled Biological Environment: Application towards Long-Lasting Neural Prostheses †

**DOI:** 10.3390/s24113619

**Published:** 2024-06-04

**Authors:** Jordan Roy, Umme Tabassum Sarah, Gaëlle Lissorgues, Olivier Français, Abir Rezgui, Patrick Poulichet, Hakim Takhedmit, Emmanuel Scorsone, Lionel Rousseau

**Affiliations:** 1 ESYCOM Laboratory for Electronics, Communication and Microsystems, CNRS UMR 9007, F-77454 Marne-la-Vallée, France; jordan.roy@esiee.fr (J.R.); sarah.ummetabassum@esiee.fr (U.T.S.); gaelle.lissorgues@esiee.fr (G.L.); olivier.francais@esiee.fr (O.F.); abir.rezgui@esiee.fr (A.R.); patrick.poulichet@esiee.fr (P.P.); hakim.takhedmit@univ-eiffel.fr (H.T.); 2 Diamond Sensors Laboratory, CEA-LIST, F-91190 Gif-sur-Yvette, France; emmanuel.scorsone@cea.fr

**Keywords:** polycrystalline diamond, neural prosthesis, impedance spectroscopy, accelerated aging, passivated layer, electrodes, bio-interfaces

## Abstract

This paper demonstrates, for the first time, the stability of synthetic diamond as a passive layer within neural implants. Leveraging the exceptional biocompatibility of intrinsic nanocrystalline diamond, a comprehensive review of material aging analysis in the context of in-vivo implants is provided. This work is based on electric impedance monitoring through the formulation of an analytical model that scrutinizes essential parameters such as the deposited metal resistivity, insulation between conductors, changes in electrode geometry, and leakage currents. The evolution of these parameters takes place over an equivalent period of approximately 10 years. The analytical model, focusing on a fractional capacitor, provides nuanced insights into the surface conductivity variation. A comparative study is performed between a classical polymer material (SU8) and synthetic diamond. Samples subjected to dynamic impedance analysis reveal distinctive patterns over time, characterized by their physical degradation. The results highlight the very high stability of diamond, suggesting promise for the electrode’s enduring viability. To support this analysis, microscopic and optical measurements conclude the paper and confirm the high stability of diamond and its strong potential as a material for neural implants with long-life use.

## 1. Introduction

Implantable neuroprosthetic devices hold the potential to restore neurological function in people with disabilities. Experiments have shown that an array of microelectrodes inserted into the cortex can capture brain activity and trigger movements in prosthetic limbs [1,2] or elicit certain visual sensations through electrical stimulation [2,3]. In such applications, the durability and stability of the device are crucial in ensuring consistent functionality over time [4]. However, over the long term, the polymers used in the device may swell and allow moisture to seep in, leading to a degradation in performance and ultimately reducing its lifespan [5,6]. This has been shown as a major hindrance to the adoption of this technology for human health, especially considering the medical implications of open brain, spinal cord and peripheral nerve surgery regarding the use of soft implantable neuroprostheses, each showing specific constraints. While several electrode designs and materials exist, they all show limitations in their usable lifetime [7]. It is thus necessary to develop functional electrodes that are as biologically inert and long-lasting as possible, to reduce the need for replacement [8].

One solution is to envision an implant based on diamond material [9,10]. Nanocrystalline diamond is known as an advantageous material for electrochemistry and biosensing [11,12]. In the last decade, it has demonstrated impressive biocompatibility, making it one of the most promising materials for living tissue interfacing and particularly for neural interfacing [13]. Indeed, diamond, as a crystalline form of carbon, shows low toxicity towards living cells and offers great adherence to cells, facilitating its integration with tissue. Diamond also does not trigger inflammatory responses and has no natural oxides, a property that few other non-toxic materials share. Typical implant materials such as silicon, polyimide and parylene only share some of these properties, which impacts their performance over time [5,8,14,15,16,17].

Diamond’s biocompatible nature has been thoroughly researched for many years [14,18,19,20,21,22,23], coupled with its semiconductor properties, allowing targeted functionalization [24]. This makes diamond an exceptional implant material.

**Figure 1:**
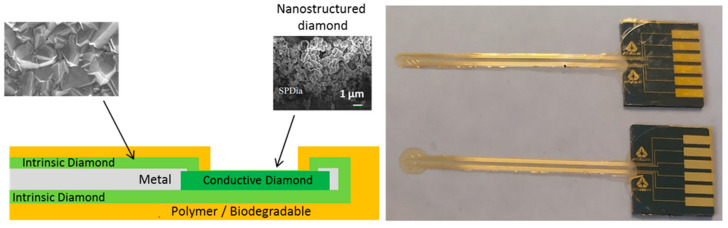
(**Left**) Schematic representation of our full-diamond electrode. (**Right**) Full-diamond implant.

Thus, in order to address the challenge of neuronal implants with a long lifetime (several years), a novel, full-diamond implant has been developed using a microfabrication process. It combines conductive diamond (boron-doped diamond (BDD)) for electrodes in contact with biological species and intrinsic diamond for full passivation (Figure 1) [25]. This passivation layer is embedded within a conductive layer (titanium nitride) in order to define the electrical access path and impede its resistivity. 

This prosthesis shows promising properties in terms of biocompatibility and hermiticity thanks to the strong bonding between the intrinsic and conductive diamond, as well as its mechanical properties. Indeed, at micrometer scales, a thin layer of diamond shows appropriate flexibility for biological uses. Here, the thickness of the diamond implant is in the range of 3 µm. This structure is coated with a polymer to easily manipulate the device (total thickness around 15 µm) (Figure 1).

This implant has been tested on the visual cortices of rodents at the Paris Vision Institute and EPFL. The visual evoked potentials (EVP) were recorded after light stimulation of the contralateral eye. The results are very encouraging [26], showing suitable functionality for the stimulation or recording of neuronal activity.

However, the question of the long-term stability of the prosthesis requires further analysis. This paper focuses on this crucial point. In this context, the demonstration is based on the aging of an interdigitated electrode (IDE) with full-diamond insulating passivation, as a representative of the actual prosthesis, which is possible thanks to its full-diamond design. 

The in vivo evaluation of electrodes requires highly skilled medical practitioners as well as the use of extensive facilities and equipment for a long period of time, namely the period for which it is necessary to evaluate the electrode’s aging. In this particular case, it must be examined over a period of 10 years. This is not ethical or practical. 

Thus, impedance monitoring is used here to evaluate changes in the diamond layer over time. For long-life testing, a thermally controlled biological environment has been designed and used for aging acceleration. For a comparative study, samples using a polymer instead of diamond have been used. 

## 2. Materials and Methods Used for Longevity Test and Aging Monitoring

### 2.1. Passivation Layer as an Aging Reference

As the outer surfaces of the prosthesis are entirely covered in diamond, its stability can be derived from that of the diamond itself, using the same manufacturing process. An aging-specific design has been developed to evaluate the coating material’s hermeticity in the test device. An IDE structure is used instead of a simple electrode as a test device to access the resistive and capacitive behavior over various frequencies through impedance analysis tools. A titanium nitride (TiN) IDE entirely coated (passivated) with intrinsic diamond, using the same fabrication process as for the previously described prosthesis, was fabricated.

Indeed, an electrode would show different impedance according to whether it is isolated from a conductive medium or not, allowing the evaluation of the insulation layer.

### 2.2. Design and Fabrication of Test Structures

**Figure 2:**
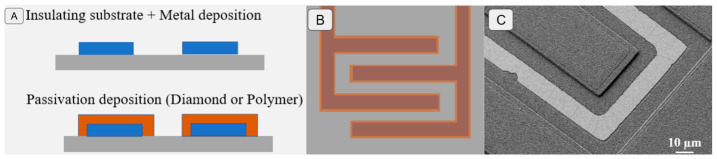
(**A**) Schematic representation of the test IDE electrode layers. (**B**) Schematic representation of the test electrode design. (**C**) Microscopic observation of a fabricated electrode.

In order to test the aging of the diamond coating and its insulating performance compared to classical polymers, two types of samples were created based on an IDE passivated with (i) intrinsic polycrystalline diamond or (ii) a polymer (SU8) (Figure 2). 

The IDE was fabricated on silicon wafer with a 1.5 µm silicon dioxide layer. For the metallic layer, a titanium nitride (TiN) layer was sputtered on the wafer. By photolithography, the IDE was patterned and the TiN layer was etched in a liquid solution (TBR 19 Technic Inc., Cranston, RI, USA). Concerning the IDE with polymer passivation, we covered the TiN IDE with a 2 µm SU8 layer (SU8 2002).

For the IDE with diamond passivation, intrinsic diamond was grown on a diamond nano-seeded substrate with a microwave plasma-enhanced chemical vapor deposition (MPECVD) reactor (SDS6K) from the SEKI company (Tokyo, Japan). We grew the intrinsic diamond at the power of 3 kW at a pressure of 30 mTorr and a temperature of 780–900 °C, with a mixture of hydrogen and methane gas. In these conditions, we grew a 2 µm diamond layer. To grow diamond, it is necessary to prepare wafers by seeding diamond nanoparticles. After this step, we deposited an aluminium nitride (AlN) layer, and, by photolithography, this layer was patterned with AlN where it was desired to grow the diamond. After diamond growth, the entire AlN layer was etched in a liquid solution and the wafer was cleaned.

For the IDE design (Figure 2B), the finger parameters were as follows:

- SU8_100 and SU8_50 were IDEs with 100 or 50 fingers covered with SU8;
- Dmd_50 and Dmd-25 were IDEs with 50 or 25 fingers covered with intrinsic diamond;
- All IDEs had the same size—a finger width of 30 µm, with spacing of 20 µm, and a length of 4.66 mm.

**Figure 3:**
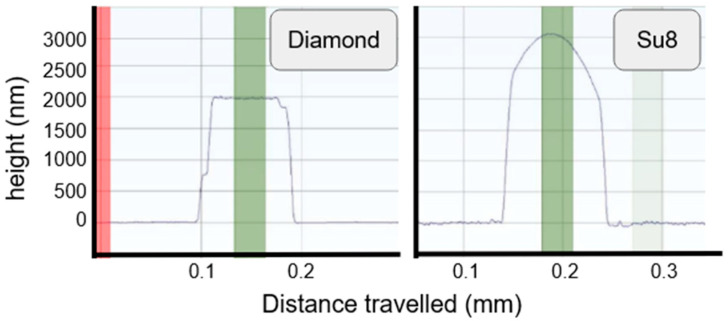
Fabricated layer profile for thickness measurements.

The results obtained for the IDE are presented in Figure 2C. The thickness measurement of the passivating layer was 2 µm for diamond and an average of 2.5 µm for SU8, with a maximum value of 3 µm (Figure 3). 

Sample parameters for labeling are presented in Table 1.

### 2.3. Aging Analysis

In chemical kinetics, Arrhenius’ law is used to link the rate at which chemical interactions occur in relation to the temperature [27,28]. This method has been extensively used for many years to evaluate the aging of many objects, including electrodes [29].

Arrhenius’ law postulates that higher temperatures increase the rate of the reaction, thus increasing the rate of electrode degradation. This relation can be used to calculate the coefficient of acceleration (k) in relation to the difference between the operation temperature and elevated temperature (T).

(1) $$k=A\cdot e^{\frac{-E_{a}}{RT}}$$

where k: coefficient of acceleration, $E_{a}$: activation energy, A: frequency factor, R: gas constant, T: temperature in Kelvins.

A and $E_{a}$ are empirical parameters determined through the comparative measurement of samples aged under different temperatures. Following previous work on the aging of bio-implants [29,30,31], the ASTM guidelines [32] provide values for the typical evaluation of such systems in the form of $k=Q^{\frac{\Delta T}{10}}$ with a conservative value of Q=2. These values would give an acceleration factor of 19.03. Considering a chemically isolated aging medium, limited testing allowed the empirical determination of an acceleration factor of 12.9 in the conditions presented in this study.

### 2.4. Impedance Analysis

**Figure 4:**
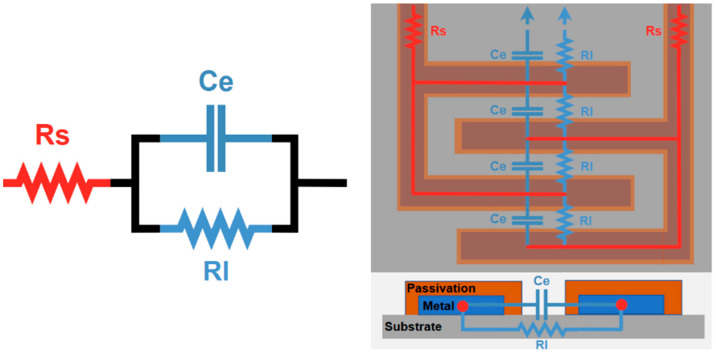
(**Left**) Simplified electrical model of the device. (**Right**) Schematic representation of the test pseudo-electrodes and their correlations with the model’s parameters.

To evaluate the material’s degradation, an analytical model of the device, based on the electrical behavior, was developed in relation to the electrode’s geometry. The model was reduced to three passive components to reflect the main electric behaviors [33] of the device (Figure 4):

(i) Rs represents the deposited metal’s resistivity’
(ii) Ce is the isolation between the conductors; since it includes the electrodes, it depends on the evolution of the electrode geometry;
(iii) Rl represents the leakage currents through the substrates or between the electrodes.

Considering Ce’s expected capacitance, with a constant medium of permittivity ($\varepsilon_{0}\varepsilon_{r}$) and an equivalent distance between the electrodes (d), its variations are mainly due to changes in the IDE’s surface area (A): $Ce=\varepsilon_{0}\varepsilon_{r}\frac{A}{d}$.

To take into account the statistical distribution of the capacitance over the IDE’s surface, a constant phase element (CPE) has been introduced as a model for Ce. In this case, the impedance associated with Ce is $Zce=\frac{1}{{Ce(j\omega )}^{\beta }}$.

The value variation of β can be seen as a reflection of the charge distribution along the different electrodes and the surface roughness. For a better interpretation, Ce can be considered as a fractional capacitor, which offers a means to evaluate charge distributions as an indicator of the surface conductivity variation [34,35].

The described transfer function is defined in Equation (2):

(2) $$Z(tot)=Rs+\frac{1}{\frac{1}{Rl}+{\left(j\omega Ce\right)}^{\beta }}$$

The measurement setup is automated using a dedicated Python 3.7 script to allow the analysis of the changes in the parameters (impedance, temperature, time, etc.) over time. The data collected are then adapted using VBscript 5.6 and the parameters are finally extracted and plotted using Matlab R2021a. Lastly, fitting is automated and supervised using the least-squares method.

### 2.5. Aging Experimental Setup with Accelerating Behavior

**Figure 5:**
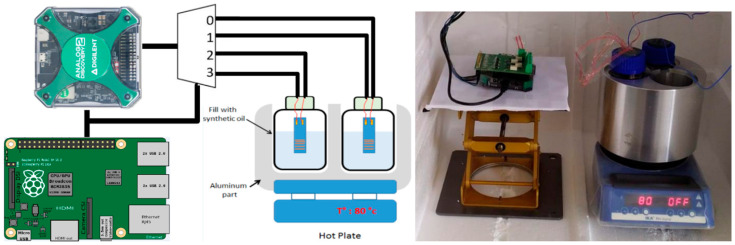
(**Left**) Schematic representation of the test setup. (**Right**) Test bench setup with samples.

To test the stability of the sample, an accelerating long-life setup has been fabricated. Figure 5 shows the setup based on an aluminum block (for thermal stability) placed on a hotplate. The block contains three tanks where a jar can be inserted. To ensure good thermal conductivity, each tank is filled with mineral oil. Each jar is filled with PBS solution and 6 samples can be placed in each one. To measure the temperature of the solution, thermocouples are inserted into the jar. The temperature of the PBS is maintained at 78 °C to ensure the acceleration of the aging according to the law of Arrhenius [29,36,37], as seen previously. 

The whole system is placed inside a Styrofoam box to protect it from the environment and ensure good thermal insulation from the outside. To check the integrity of the sample, impedance measurements are conducted regularly (every hour) with a large frequency range, from 100 Hz to 10 MHz. The results are coupled with an analytical model for the monitoring of the implants’ aging over time. The system also allows the stimulation of the electrode strip via current injection. The current stimulation mimics the behavior of the implant during artificial spiking activity (induced by stimulation).

## 3. Results

The collected data consist of four samples: two, denoted SU8_100 and SU8_50 (Table 1), are associated with the polymer passivation layer, and the two others concern the diamond passivation layer, labeled Dmd_50 and Dmd_25 (see Table 1). The data include a total of 7009 impedance spectra (modulus and phase) over 100 frequency points logarithmically spaced between 100 Hz and 15 MHz, with a measurement occurring every hour for each sample. This represents continuous recording for 39 weeks under accelerated aging conditions. 

Considering that there are 7009 measurements for each electrode, data analysis is required for the interpretation of the results. Thus, for a better understanding, further visual analysis is performed on a single frequency point considered representative of neural activity recording: 1 kHz. Parameter evolution is extracted using automated supervised least-squares fitting.

### 3.1. Stability Comparison

**Figure 6:**
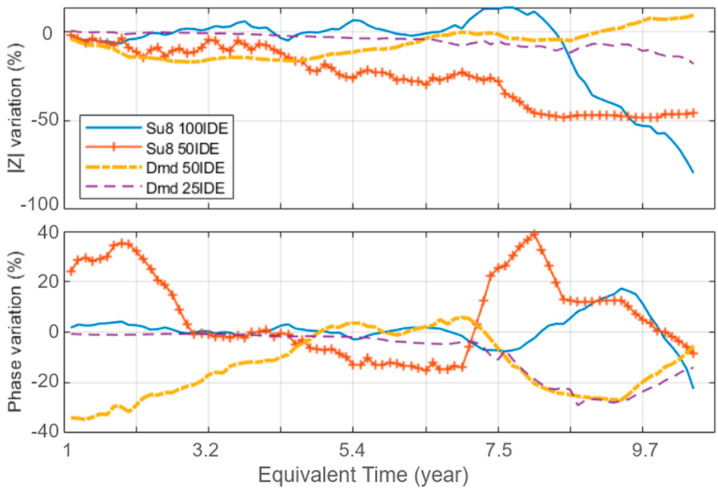
Extracted impedance variation (modulus and phase) for each sample design at 1 kHz over time.

To evaluate the stability of the pseudo-electrodes over time, the evolution of each sample’s impedance modulus and phase at 1 kHz is considered, as in Figure 6 (dotted lines refer to diamond; continuous lines refer to the SU8 polymer). The values presented are normalized compared to the first measurement. Our tests show different aging changes and potential failure modes for each material. At first consideration, the diamond passivation layer shows higher insulation properties, represented by the mean |Z| value over time, presented in Table 2. The diamond passivation also shows more stable behavior compared to the SU8 layer, which presents some failure, which is visible as a high modulus and phase variation, potentially indicating the occurrence of a distinct failure.

The diamond sample’s phase shows a synchronous variation after an equivalent aging time of 7.5 years of comparable amplitude, indicating a common cause. This effect seems to be correlated to the acceleration of the degradation of the SU8 samples, which may have altered the PBS medium.

**Figure 7:**
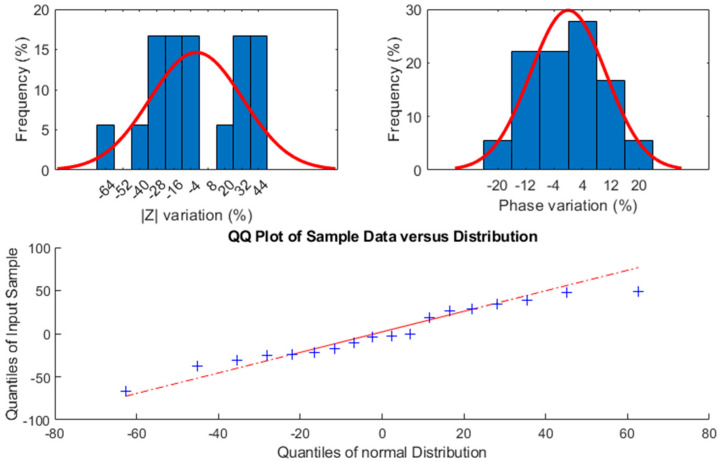
Impedance variations of diamond electrodes over 23 years of equivalent aging time on 6 sets of 3 electrodes.

To evaluate the reproducibility, several sets of diamond electrodes have been separately aged for a longer period, to extract the statistical variance from the process. The results can be observed in Figure 7. Although these samples have not been monitored over time, their final impedance values at 1 kHz after being aged together show two main aging behaviors: one set increased in modulus, as the Dmd_50 sample previously showed, while the second set progressively decreased, as Dmd_25 did. Further studies are needed to analyze the reasons for such differences as no obvious pattern emerged. One sample failed with a −64% modulus variation, which may have been due to an error in this particular sample’s preparation as the wire connections were found to be exposed to the medium. This sample has, nevertheless, been retained in our data.

### 3.2. Model’s Parameters’ Evolution

**Figure 8:**
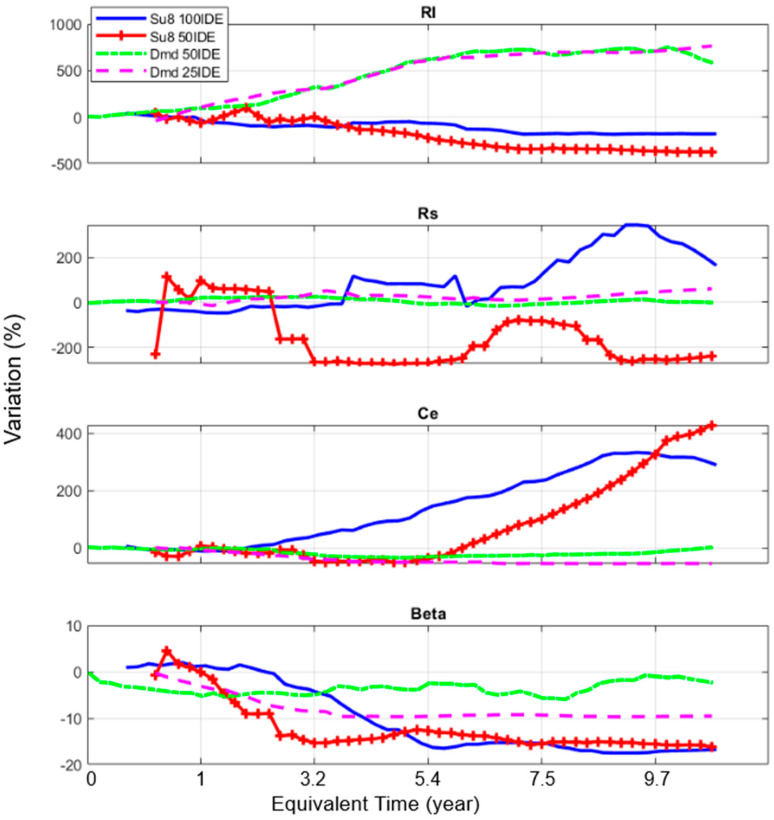
Evolution of the variations in the extracted model parameters for the SU8 and diamond samples as a function of the equivalent time.

For each material and spectrum, the extraction of the model parameter values was performed, and this allowed the monitoring over time of the degradation occurring within the samples. Figure 8 shows the side-by-side comparison of the Rs, Rl, Ce and β parameters’ evolution throughout the experimental duration.

The capacitance of polymer samples tends to increase over time, which may be due to an increase in their surface, eventually becoming porous, which reflects the degradation of the passivation layer. Rl and β seem to offer some indications on this matter, as the current leakage increases for the polymer layer (Rl decreases), while the charge distribution (β) shows imperfect capacitive behavior (β decreased). β is also related to the roughness of the surface; a decrease in β over time can be associated with an increase in the roughness value. As such, it is inferred that, over time and successive measurements, the polymer passivation tends to degrade and leak.

Comparatively, after an initial stabilization period, the diamond samples show relatively stable capacitance values over time. This is confirmed by the stability of the β parameter, showing very little variation over time, also confirming the stability of the surface electrode capacitance Ce. The Rl value tends here to increase at first until reaching a plateau, in synchronization with Ce. This indicates that the diamond passivation starts in a less conductive state before stabilizing, meaning an increase in its electrical insulation behavior. It can be assumed that the diamond layer is in a more hydrogenated state after growth, which stabilizes as the hydrogen reacts [38,39].

This comparison, across more than nine months of experiments under accelerated aging conditions, highlights the very good stability of diamond as a passivation layer compared to the polymer material (SU8). Indeed, its capacitance and charge distribution show high stability over time, indicating no loss in functionality, besides an initial settling time due to the fabrication process. This confirms the potential of using diamond as an electrically stable layer for biomedical devices.

### 3.3. Microscopic Observation

**Figure 9:**
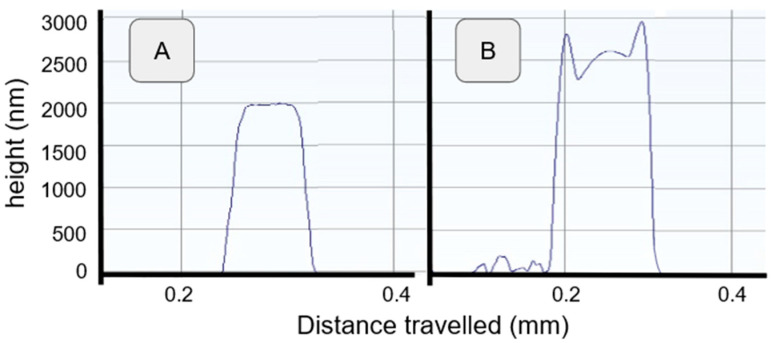
Thickness profile comparison before and after aging. (**A**) Dmd_50 after aging. (**B**) SU8_50 after aging.

To correlate the previously shown analytical results, a sample analysis has been performed, starting with profilometer measurements (Figure 9), which can be compared with Figure 3. The diamond samples retain their original shape, indicating no loss in structural integrity, while the SU8 samples show distortion and a diminished profile, indicating the clear instability of the passivation layer.

**Figure 10:**
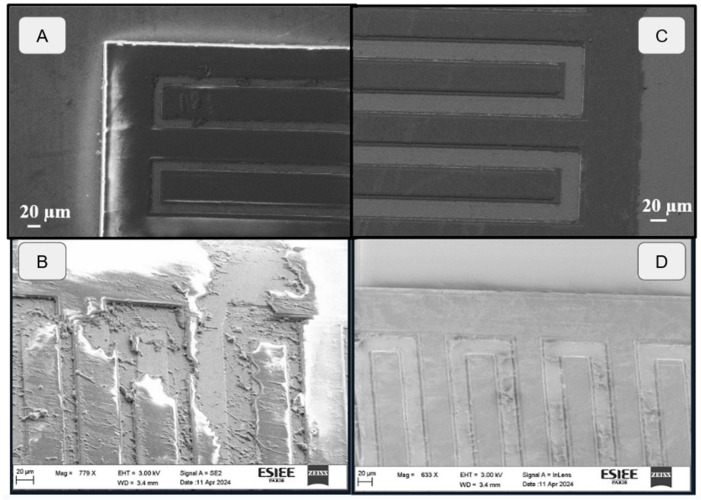
Microscopic and SEM observation of IDE’s surface before and after aging. (**A**) SU8_50 before aging. (**B**) SU8_50 after aging. (**C**) Dmd_50 before aging. (**D**) Dmd_50 after aging.

**Figure 11:**
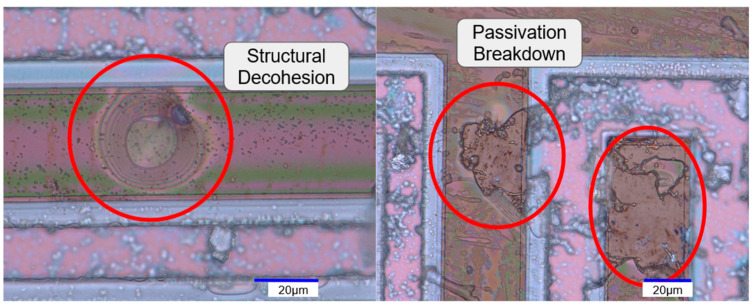
Optical microscopic observation of SU8_50’s surface after aging with a focus on different failure types.

Several more observations have been made using both microscopic and SEM observation, as shown in Figure 10, where the comparison before and after aging shows a clear passivation failure for SU8. The diamond layer has retained its original shape, further confirming its structural integrity, although some undefined particles appear lodged between the IDE’s elements. These particles are supposed to originate from the SU8 sample’s degraded layers, as previously mentioned during the analysis. The high density of these particles on the SU8 samples, and near their degraded parts in particular, further increase our confidence in their origin, without leading to a conclusion (Figure 10 and Figure 11).

A further analysis of the SU8 passivation failures shows the progress of the localized structural decohesion of the passivation, as seen in Figure 11. This delamination seems to further progress until the SU8 layer’s structure breaks, exposing the inner layers. These effects accumulate over the electrode’s surface, explaining the relatively high impedance variations compared to diamond, as discussed previously. For the diamond samples, no delamination was optically found.

## 4. Discussion

This work serves to demonstrate that the diamond material will be the next-generation material for long-term medical implants. With its intrinsic properties, carbon interface, lack of oxidation, high stability and biocompatibility, the diamond material shows great promise for the medical field. To validate these assumptions, this study of IDE structures provides a complete analysis of the aging characteristics of the material and an analysis on a simple device. This choice was made to allow a fine comparison between the bioimpedance measurements over time under specific aging conditions and to obtain analytical models expressing several significant parameters, such as capacitance, resistance or charge variations. Our analytical model, focusing on a fractional capacitor, provides clear insights into the surface conductivity variation, and the presented results highlight the superior stability of diamond as a passivation layer compared to polymer materials like SU8.

The next step will be to complete the aging setup with additional varying conditions, such as temperature ramps, pH cycling or mechanical stress, with samples in separate media to avoid potential cross-contamination. A deeper analytical model coupled with micro-photography monitoring could allow the extraction and validation of more parameters, paving the way towards a deeper understanding of the material’s properties and improving our initial predictions. Our methodology has proven its efficiency and will be extended to converge towards an improved and fully characterized neuro-implant.

Furthermore, to validate the attractiveness of diamond materials for medical applications, a comparison between different materials is planned, such as silicon nitride or silicon dioxide as a passivation layer in an accelerated long-life setup, as well as validating the stability of conductive diamond as an interface for sensing, while recording or stimulating neuronal activity. These studies will result in a full-diamond implant that combines conductive and intrinsic diamond for long-life use in medical devices. Thanks to this development based on diamond materials, the production of medical implants that are stable throughout a patient’s life can be envisaged. 

The sample’s fabrication is based on a completely new manufacturing process based on MPCVD diamond growth on 4-inch wafers and is fully compatible with standard cleanroom microfabrication and commercial equipment [26]. Therefore, the production of diamond-based neural implants will be directly transferable, and previous projects have already conducted some diamond implant manufacturing [40]. Furthermore, today’s microelectronics sector is investing widely in this material for the development of new applications in MEMS sensors [41] or high-energy devices [42,43] as examples. Thus, in the near future, it is expected to become possible to easily produce diamond layers on larger wafers, thus multiplying the production output of such devices.

Introducing a new material for medical application requires many years of research and regulatory processes; as such, it is necessary to use animal experiments to validate the innocuity of the material. In the European project NEUROCARE, several preliminary studies have been performed with diamond samples in animals to validate their biocompatibility [44], but it will be necessary to continue these experiments in a future project to accelerate the transfer of this technology to the medical field. 

## 5. Conclusions

This study aimed to assess, for the first time, the long-term stability and aging characteristics of intrinsic polycrystalline diamond as a biocompatible coating material for in vivo implants.

This investigation was based on an electric impedance analysis of diamond-passivated IDE samples compared to polymer-coated IDE samples. The results obtained demonstrated evolving impedance patterns over time, revealing information about the behavior of the test devices and therefore the materials. The key parameters concerning electrical insulation were clearly stable for the diamond compared to the polymer, where failures appeared during the experiments. Accelerated aging over an equivalent time of 12 years with thermal activation was used to obtain these results.

The impedance analysis of isolated test electrodes, coupled with the developed analytical model, provides valuable insights into the material efficiency of full-diamond electrodes at a low cost and with ease of use. The consideration of Ce as a fractional capacitor has allowed the evaluation of the charge distributions as indicators of the surface conductivity variation and behavior, offering a better understanding of diamond’s aging dynamics while used as a passivation layer.

This work demonstrates the strong interest in diamond materials for the in vivo long-term use of neuronal implants.

## Figures and Tables

**Table 1:** Samples fabricated and their dimensions.

	SU8_100	SU8_50	Dmd_50	Dmd_25
Conductor	TiN (200 nm)			
Passivation material	Polymer	Polymer	Diamond	Diamond
Passivation thickness	2 µm to 3 µm			
IDE arrays	100	50	50	25

**Table 2:** Sample parameters’ evolution over time, including initial transient period.

	SU8_100	SU8_50	Dmd_50	Dmd_25
Passivation	Polymer	Polymer	Diamond	Diamond
Mean |Z| @1 kHz	5.2 kΩ	6 kΩ	15 kΩ	35 kΩ
Module variation	±51.1%	±27.6%	±14.1%	±9.8%
Phase variation	±24.8%	±29.4%	±14.3%	±9%
Rs variation	±104.4%	±135.4%	±47.9%	±34.3%
Rl variation	±58%	±115.8%	±28%	±37%
Ce variation	±71%	±136.4%	±24.9%	±35.5%
Stabilized Ce	1.15 µF	524 nF	540 nF	356 nF
Equivalent stabilized Ce surface	14.2 µm^2^	6.5 µm^2^	6.7 µm^2^	4.4 µm^2^
β variation	±12.1%	±14.2%	±2.9%	±5.2%
−30% functionality (equivalent time)	6.4 years	8.1 years	∅	∅

## Data Availability

Data are contained within the article.
